# Therapeutic response to corticosteroids in a critically ill patient with COVID-19: A case report

**DOI:** 10.1097/MD.0000000000021597

**Published:** 2020-07-31

**Authors:** Kaige Wang, Fen Tan, Rui Zhou, Dan Liu, Zhong Ni, Jiasheng Liu, Fengming Luo

**Affiliations:** a Department of Pulmonary and Critical Care Medicine, West China Hospital, Sichuan University, Chengdu; b Department of Pulmonary and Critical Care Medicine, The Second Xiangya Hospital, Central South University, Respiratory Disease Research Institute of Hunan Province, Changsha; c Department of Gastrointestinal Surgery, Renmin Hospital of Wuhan University, Wuhan, China.

**Keywords:** corticosteroids use, critically ill patient, COVID-19, SARS-CoV2

## Abstract

**Introduction:**

Since the coronavirus disease 2019 (COVID-19) outbreak in Wuhan in late 2019, controversy on the use of corticosteroids for COVID-19 has obtained increasing attention. We present 1 critically ill patient who had a rapid therapeutic response to moderate-dose corticosteroids.

**Patient concerns:**

A 53-year-old critically ill woman from Wuhan suffered with COVID-19.

**Diagnosis:**

The chest computed tomography scan was suggestive of COVID-19. The diagnosis was confirmed by a real-time reverse transcription polymerase chain reaction test for SARS-CoV-2. The critically ill status was characterized by worsening dyspnea, progressing bilateral lung consolidation, and poor oxygenation (SiO_2_/FiO_2_:110 mm Hg).

**Interventions:**

The patient was treated with a moderate dose of intravenous corticosteroids and high-flow nasal cannula oxygen therapy.

**Outcomes:**

After the initiation of corticosteroids, the patient rapidly improved over the following 6 days. Serial chest computed tomography scans showed good absorption of the consolidations. The patient was discharged on Day 17 of hospitalization without obvious adverse effects.

**Conclusions:**

Early use of moderate-dose corticosteroids over a short period may enhance recovery from COVID-19 in critically ill patients.

## 1 Introduction

Since the coronavirus disease 2019 (COVID-19) outbreak began in Wuhan in December 2019, COVID-19 has become pandemic, with more than 8 million laboratory-confirmed cases by June 22, 2020.^[1]^ According to early reports from China, 16% of hospitalized patients infected with severe acute respiratory syndrome coronavirus 2 (SARS-CoV-2) experience severe disease,^[2]^ and of 17% to 29% of patients hospitalized with SARS-CoV-2 infection has been reported to develop acute respiratory distress syndrome (ARDS).^[3,4]^ There is currently no targeted antiviral treatment for COVID-19. Supportive care is provided to help relieve symptoms and protect organ function. According to the opinions of some experts,^[5]^ corticosteroids should not be used in patients with SARS-CoV-2-induced lung injury or shock. Recently, the results of a clinical trial in the UK show that low dose dexamethasone can reduce the mortality of COVID-19 patients with mechanical ventilation by about one third.^[6]^ However, many clinicians have a different perspective, based on their clinical experience.^[7]^ We report a case of a critically ill patient with COVID-19 pneumonia who recovered after corticosteroid therapy. This case illustrates the potential benefits of corticosteroid therapy for COVID-19. The report was approved by RHWU Research Ethics Committee (WDRY2020-K068). The patient has provided informed consent for publication of the case.

## 2 Case report

**Figure 1:**
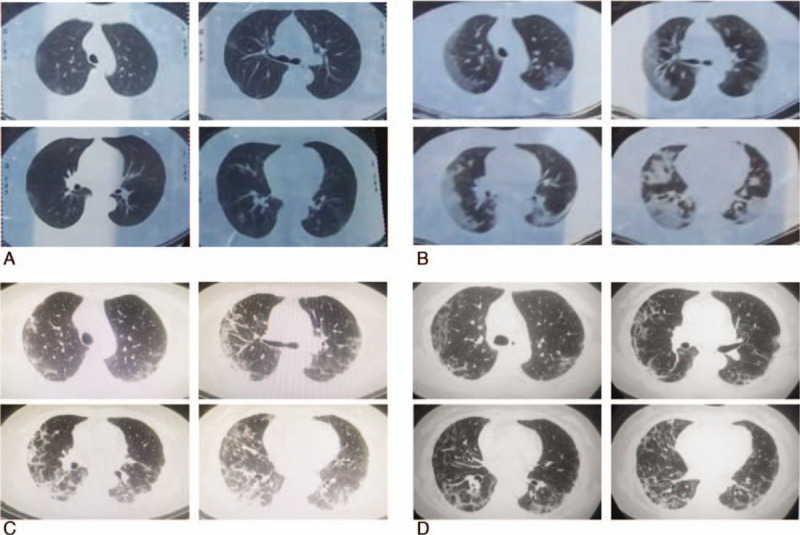
Serial chest computed tomography images over the course of the illness. A, Day 2: Ground-glass opacities are scattered peripherally in both lungs. B, Day 8: There is diffuse bilateral consolidation of the ground-glass opacities in both lungs. C, Day 18: The computed tomography (CT) image reveals partial resolution of the lung consolidation observed in the previous CT scan on Day 8. D, Day 23: The CT scan reveals almost complete resolution of the lung consolidation.

A 53-year-old woman living in Wuhan, China was admitted to a designated COVID-19 hospital because of fever and cough. The fever had started 1 week previously without obvious cause, and her highest recorded body temperature was 38.4°C. She also had a dry cough without chest pain, hemoptysis, or diarrhea. Her initial chest computed tomography (CT) (Fig. 1A) showed ground-glass exudative lesions scattered in both lungs. The test for SARS-CoV-2 infection by real-time reverse transcription polymerase chain reaction (RT-PCR) assay of oropharyngeal swabs was negative. She was initially treated with oseltamivir in outpatient department. However, her condition worsened, and developed dyspnea, requiring designated wards hospitalization. She had the history of hypertension with long-term administration of amlodipine.

On presentation, her temperature was 38.3°C. Her other signs were: respiratory rate 28/min; SiO2/Fio2 170 mm Hg; body weight 68 kg; heart rate 106/min; blood pressure of both arms 108/ 70 mm Hg. Cardiovascular examination revealed tachycardia with regular rhythm, normal first and second heart sounds, and no murmurs, gallops or rubs. On auscultation of the lung fields, breath sounds were coarse with wet rales scattered at both lungs. Her abdomen was soft and non tender with no palpable organomegaly. Neurological examination did not reveal any focal neurological deficit.

On hospitalization, her whole blood cell count showed neutrophilia, and lymphopenia. She had a markedly elevated C-reactive protein (CRP). The detailed information and the change in the whole hospital course are shown in Table 1. The test for COVID-19 infection by RT-PCR assay was positive. Additional laboratory parameters including alanine aminotransferase, aspartate aminotransferase, and creatinine levels were normal. Procalcitonin, G-test, GM-test, and antibody against influenza A virus and influenza B virus were negative, as well as antineutrophil cytoplasmic antibody and antinuclear antibody. Repeated chest CT showed progressive consolidation in both lungs (Fig. 1B). The patient was laboratory confirmed COVID-19. After admission, we treated him with antiviral (arbidol and thymosin α1) and oxygenation supportive with high flow nasal cannula for 2 days. However, on Day 3, the patient dyspnea worsened rapidly. Her respiratory rate was increased to 32/min, PaO_2_/FiO_2_ decreased to 110 mm Hg. The patient refused noninvasive ventilation and mechanical ventilation. We then treated him with corticosteroids (80 mg twice a day for 3 days, then 40 mg twice a day for the following 3 days), with the patient's consent. The detailed treatment is shown in Table 2. Inflammatory makers including whole blood cell count and CRP shown in Table 1 and the record of parameters of respiratory status overtime shown in Table 2 illustrated the patient's improvement after therapy. Repeated CT scan indicated consolidations were absorbed partly on Day 11(Fig. 1C) and improved obviously on Day 16 (Fig. 1D). Repeated detection for SARS-CoV-2(performed twice) through oropharyngeal swabs was negative. The patient recovered without any obvious adverse effect and discharged on Day 17.

**Table 1:**
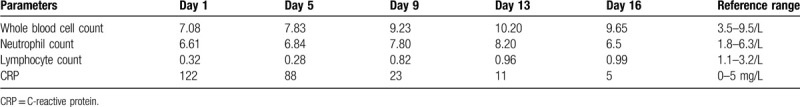
Change of the whole blood cell count and CRP in the hospitalization.

**Table 2:**
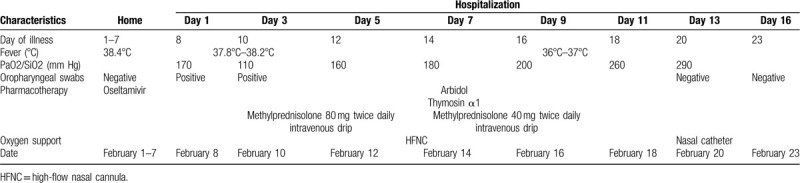
Temperature, blood oxygenation level, detection for nucleic acid of nCoV-2019, and treatment on day of illness and day of hospitalization, February 1 to February 23, 2020.

## 3 Discussion

This patient living in Wuhan with respiratory symptoms was suspected of COVID-19 by early chest CT scan. Their RT-PCR assays in respiratory specimens confirmed the diagnosis. Negative of procalciton in test, G-test, GM-test indicated no bacterial or fungal infection. Dyspnea rapidly worsened in the course of nonspecific antiviral treatment and symptomatic treatment. The chest CT showed progressing consolidation of the lung. According to Kigali Modification of the Berlin Criteria,^[8]^ the patient was diagnosed as moderate ARDS. Upon condition worsened to critically illness, the patient was treated with moderate dose use of corticosteroids for 6 consecutive days. The condition improved significantly illustrated by the symptoms and respiratory parameters as well as chest CT. Finally, tracheal intubation was avoided.

On the corticosteroids use in COVID-19, experts held different opinions. According to the observational studies on corticosteroids treating patients with severe human coronavirus including SARS-CoV and Middle East respiratory syndrome coronavirus, and other severe respiratory virus infections, some authors speculated that corticosteroids use could not benefit for the lung injury in COVID-19, even for the critically ill patient.^[5]^ However, physicians held a different opinion when fought with COVID-19 in front-line clinical service. Cao and colleagues recommend prudent use of the corticosteroids in the most critically ill patients.^[7]^ Furthermore, lung pathological manifestation in severe COVID-19 showed pulmonary edema and hyaline membrane formation.^[9]^ This characteristic supports timely and appropriate use of corticosteroids in critically ill patients. The consensus statement by the Chinese Thoracic Society recommends using low-dose corticosteroids (1–2 mg/kg d, 5–7 days) for critical COVID-19 patients prudently.^[10]^ The timing of application is as follows: Rapid progress in imaging (more than 50% in 24–48 hours); Under the condition of resting without oxygen inhalation, SpO2 ≤93%, respiratory distress (respiratory frequency ≥30 times/min) or oxygenation index ≤300 mm Hg. (Both conditions need to be met simultaneously.) The patient described here developed critically ill on Day 9 from the initial. A short course of corticosteroids at a moderate dose benefited the patient and prevented from the need of mechanical ventilation. The consolidation in the lung improved gradually without obvious adverse effects. Because of the impaired antibody responses existed in those given corticosteroids in the previous study,^[11]^ adverse effect will be tracked after discharge for a period.

In conclusion, early use of corticosteroids at moderate dose in short course may enhance the critically ill COVID-19 patient recovery. However, this case is a retrospective study, persuasive clinical evidence is still needed urgently.

## Acknowledgments

The authors thank the patient and his family for their kind cooperation.

## Author contributions

**Conceptualization:** Kaige Wang.

**Data curation:** Kaige Wang and Fen Tan.

**Formal analysis:** Fengming Luo.

**Funding acquisition:** Fengming Luo.

**Investigation:** Rui Zhou and Fen Tan.

**Methodology:** Fengming Luo.

**Project administration:** Kaige Wang and Dan Liu. Resources: Zhong Ni.

**Writing – original draft:** Kaige Wang and Fen Tan.

**Writing – review & editing:** Fengming Luo and Jiasheng Liu.
